# Development and Psychometric Properties of the Women’s Recovery of Postnatal Perineal Injuries Questionnaire (WRPPIQ)

**DOI:** 10.30476/ijcbnm.2020.85610.1279

**Published:** 2020-10

**Authors:** Nahid Jahani Shoorab, Ali Taghipour, Habibollah Esmaily, Robab Latifnejad Roudsari

**Affiliations:** 1 Nursing and Midwifery Care Research Centre, Mashhad University of Medical Sciences, Mashhad, Iran; 2 Social Determinants of Health Research Centre, Mashhad University of Medical Sciences, Mashhad, Iran; 3 Department of Midwifery, School of Nursing and Midwifery, Mashhad University of Medical Sciences, Mashhad, Iran

**Keywords:** Recovery of function, Perineum, Postnatal care, Questionnaire, Validity

## Abstract

**Background:**

Recovery of postnatal women with perineal injuries, especially when perineal tear is severe, occurs much later than the healthy women. There is no specific questionnaire to assess the postnatal recovery in these women. The aim of this study was development and psychometric evaluation of a new tool to measure women’s recovery of postnatal perineal injuries questionnaire (WRPPIQ).

**Methods:**

In this validation study, which was conducted based on the method developed by DeVellis (2003), 270 women with postnatal perineal injuries who referred to healthcare centers in Mashhad, Iran, were studied between 2018 and 2020. This method consisted of steps: (1) definition of postnatal recovery based on in-depth qualitative interview with 22 women, (2) generation of an item pool, (3) selection of the Likert scale, (4) review of the initial item pool, (5) inclusion of items from relevant instruments, (6) conducting exploratory factor analysis, (7) evaluation of the items, and (8) optimization of the scale length.

**Results:**

The initially generated item pool consisted of 144 items on a 5-point Likert scale, which reduced to 85 items following face and content validity measurement. The value of the SCVI/Ave was measured 0.901. The conduction of exploratory factor analysis resulted in 33 items and three factors including evidence of wellness, emotional changes as well as independence and support. The Cronbach’s alpha for the three factors was calculated 0.92, 0.80, and 0.83, respectively.

**Conclusion:**

WRPPIQ has validity and reliability to measure the women’s recovery of postnatal perineal injuries in Iran. It is, therefore, recommended that health care providers to assess women’s recovery of postnatal perineal injuries using this newly developed questionnaire.

## INTRODUCTION

Perineal injuries refer to the tears, to some extent, which occur during the childbirth in the perineum, inside the vagina or other parts of the vulva, including the labia. It is classified into four types. First-degree injuries are limited to the fourchette and superficial perineal skin or vaginal mucosa. In second-degree injuries, the perineal muscles are also damaged; in the third degree injuries, the anal sphincter and in the fourth degree injuries, the rectal wall are damaged. ^1^ Perineal injuries in up to two-thirds of cases occur during vaginal delivery. ^2^ The first 4-6 weeks after delivery has been traditionally called postpartum recovery. ^3^ Mothers’ short-term follow-up for six weeks is not enough to identify and overcome their problems, especially in those with perineal injuries. ^4 , 5^ Postnatal recovery for women with perineal injuries, especially severe cases, can take a long time after birth, but there is no specific questionnaire for health care providers to know how long the recovery after birth may take, so they should continue the postnatal care. Therefore, the mothers’ long-time sexual, urinary and defecation problems are neglected, and health care providers and midwives mainly try to evaluate the early complications such as bleeding, infection or eclampsia in routine care, and other late complications such as urinary problems are less questioned and followed-up. ^6^ Neglecting this issue threatens the maternal health in all known aspects including physical, emotional and social recovery in the postnatal period. ^6 , 7^ Although how to measure health is a major problem in clinical medicine. ^8^ Review of literature revealed that in one study, 28-item General Health Questionnaire (GHQ-28) and in another study shortened version of 36-item Quality of Life questionnaire (SF-36) were used to assess the maternal health in postpartum period. ^9^ The GHQ-28 questionnaire has not been designed specifically for women’s health. Also, none of these questionnaires has been designed for women’s postnatal health with perineal injuries. The modified Hopkins-Campbell (2008) is another questionnaire used to assess postpartum recovery and its validity and reliability have not been investigated. ^10^ Therefore, these questionnaires are not valid to assess the postnatal recovery; as a consequence, women may be deprived of adequate care. To the best of our knowledge, there is no tool which was specifically designed for these women, while the emphasis in postnatal care is continuity of care for postpartum women. ^3 , 4 , 11^ It seems that, the present study was conducted to develop and validate a questionnaire for postnatal recovery in women following perineal injuries. Therefore, it can lead to improvement of the quality of care of these women through measuring their postnatal recovery and providing appropriate and timely measures to expedite their recovery. 

## MATERIALS AND METHODS

This validation study is part of a larger sequential exploratory mixed method design, which addressed the development and validation of an instrument to measure postnatal recovery in women following perineal injuries. Validation study followed an established procedure developed by DeVellis in 2003 ^12^ in eight stages, which has been elaborated below: 

### The First Stage: Definition of Postnatal Recovery

To define the dimensions of postnatal recovery in women following perineal injuries, a qualitative study based on the conventional content analysis was conducted. The qualitative content analysis is the preferred method in cases where there is no specific theoretical framework for that phenomenon or concept, and there is little knowledge about the subject. ^13^ The target population consisted of all Iranian women who had given birth to their babies with varying degrees of perineal tears during the study.

The participants were 22 Iranian women who experienced varying degrees of perineal injuries during their childbirth, and attended Ommol-banin hospital, Mashhad, Iran and were purposefully selected from 20 April to 25 December 2016. They were included in the study if they were Iranian and attended hospital in a time period between 10 days to one year after delivery, did not experience a stressful event like divorce or death of the first degree relatives in the postnatal period or pregnancy, did not have a recognized illness, and were not drug abuser. Exclusion criterion was unwillingness to take part in the interview. 

To recruit participants, we extracted the list of eligible respondents from the maternity records and their information including national code, telephone number and address was obtained from Hospital Information System (HIS). Then, they were called and asked to participate in the study. 25 postnatal women were invited to participate in the study, but three of them refused to do so, because two women had no time for the interview and the spouse of another one did not agree with it. If the participants agreed to take part in the study, then a time and place convenient to them was arranged for the interview. It is noteworthy that during the participants’ invitation, the strategy of maximum variation was adopted and women with varying degrees of injury and at the different time points in postnatal period and also with different modes of delivery and parity were recruited. The sample size was determined based on data saturation, so that sampling was terminated when no new code was obtained during the last three interviews. 

Data were collected through face-to-face, semi-structured in- depth interviews (between 40 and 70 minutes). Interview questions consisted of: When did you feel healthy after birth? What changes did you experience after birth? Based on your experience, how did you recover after giving birth? And what health problems did you experience with perineal tears? The interviews were conducted in a quiet room in the gynecology clinic in the hospital, following signing the informed consent form by the participants. All interviews were recorded and transcribed verbatim, immediately after the interview. Data were analyzed by conventional qualitative content analysis suggested by Elo and Kingas (2008) simultaneously with data collection. ^13^ The method had three phases of preparing, organizing and reporting. In the first phase, the transcripts were read several times to obtain a general understanding of the concept; then, the related parts of the text to the postnatal recovery were identified. In the second phase, i.e. organizing, the process of coding, categorizing, and identifying of the main category of postnatal recovery was carried out. In the third phase, the emerged categories regarding postnatal recovery after perineal trauma were presented in a four-category model of recovery. The validity of the study was assessed by member check and peer debriefing. Data were organized using MAXQDA 10 software.

### The Second Stage: Generation of an Item Pool

**Figure 1:**
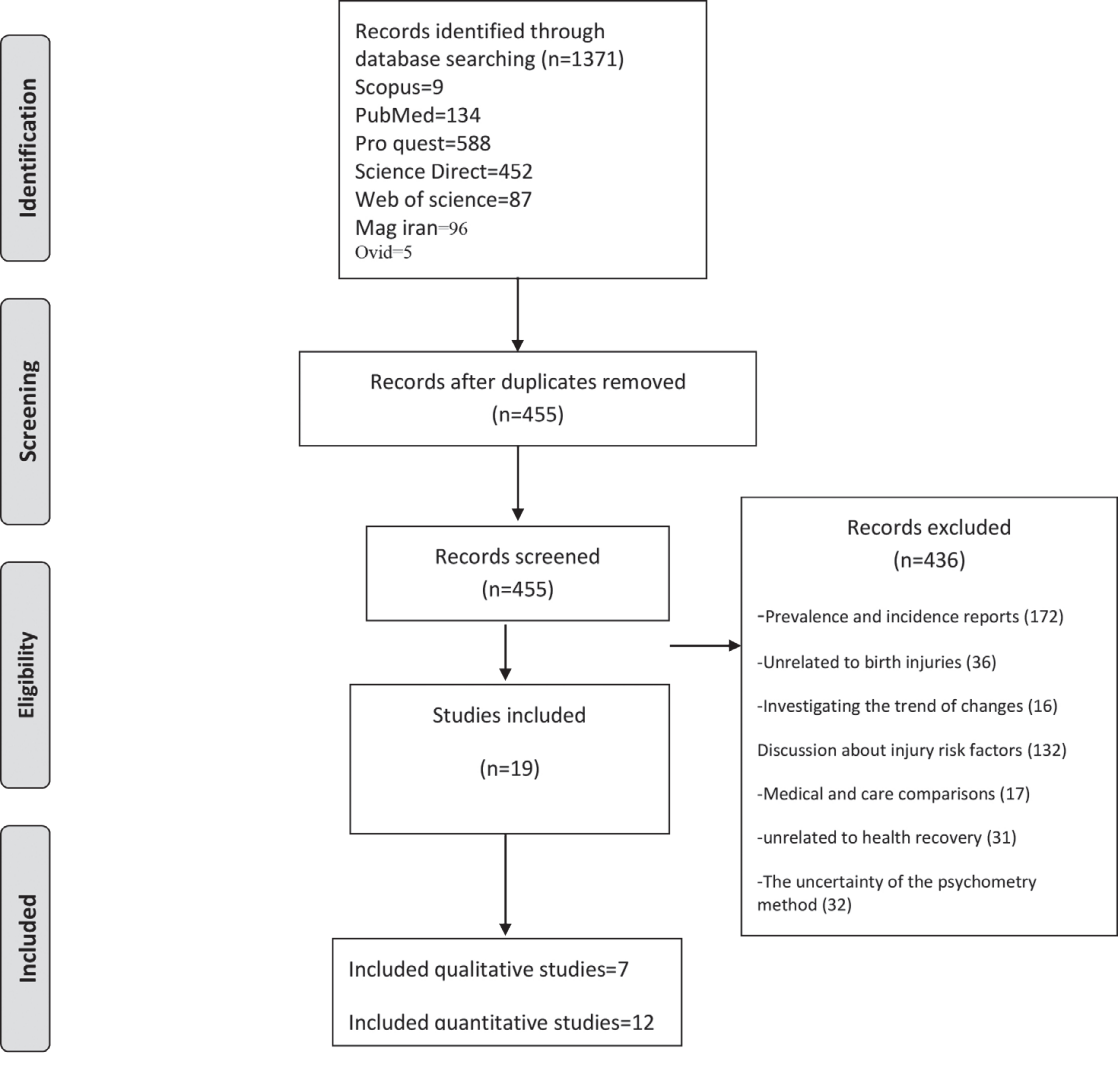
Flow chart of the literature search

The item pool was derived from qualitative data as well as literature review. For this reason, at first, the operational definition of the concept of recovery was developed based on qualitative data and then the items were generated according to the dimensions of the main concept. Literature search was also done without time limit in databases including PubMed, Ovid, Scopus, ProQuest, Web of Science, and Science Direct as well as Iranian databases of Magiran between June 2015 to January 2020, using English keywords and their Persian equivalents of: recovery, postnatal, postpartum, wellbeing, health, psychometry, validation, inventory, questionnaire, scale, tool development, instrument development Devillis, perineal tears and perineal trauma. The search strategy of the articles is shown in Figure 1.

### The Third Stage: Selecting the Likert Scale as Format for Measurement 

In the third step, since the tool was developed to measure the attitude on recovery, 5-point Likert scale was selected as the scale. 

### The Fourth and Fifth Stages

In stages four and five, reviewing the initial item pool and inclusion of items from relevant instruments was carried out.

Stages 4 and 5 were performed concurrently so that the experts could comment after adding possible items. In stage 4, the initial item pool was reviewed by the research team and the repetitive and ambiguous items were removed. 

In stage 5, three items of the Female Sexual Function Index (FSFI) was added to the item pool. Then, face and content validities of the questionnaire were measured. Face validity was measured using two qualitative and quantitative methods. To determine qualitative face validity, five participants assessed the difficulty, relevance, and clarity of the items. Thereafter, the questions were revised by the research team according to the participants’ viewpoints. To measure the quantitative face validity, the participants scored each item of the tools based on five-point Likert scale as: completely important (5), somewhat important (4), moderately important (3), slightly important (2), and not important at all (1). Then, the impact score for each item was separately calculated using the formula of Impact Score, which is equal to the percentage of the raters who scored 4 or 5 × mean score for the importance of each item. Impact Score of more than 1.5 were considered for quantitative face validity.14 In this way, three items were semantically difficult for the participants, which were corrected and their validity was confirmed thereafter. Then, the initial questionnaire with 144 items were emailed to ten experts, who preferably had clinical experience, to evaluate face and content validity. They included three experts in psychometrics, five reproductive health specialist and two gynecologists. 

To measure the content validity, we used both Content Validity Index (CVI) and Content Validity Ratio (CVR). To determine CVR, Lawshe formula and expert judgment for each item and the whole tool were used. In this formula, N is the total number of experts and ne is the number of expert(s) stating that essential. Then, the experts responded to each item of the initial questionnaire based on 3-point Likert scale (1.not necessary 2. useful but not necessary, 3. necessary). ^14^ To determine CVI, we collected the opinions of ten aforementioned experts in four-point Likert scale on each item (Score 1= not relevant, 2=slightly relevant, 3= relatively relevant to 4= completely relevant) and calculated the CVI using the following formula. 

$\mathrm{CVI}=\frac{\mathrm{Number of raters giving a rating of 3 or 4}}{\mathrm{total number of raters}}$

All items were evaluated in terms of CVI. Values above 0.79 were retained for CVI. The items with a CVI of 0.7-0.79 were discussed again with the research team and revised. The items with a CVI less than 0.7 were removed from the questionnaire. ^15^

Also, the mean of SCVI/Ave was calculated to adjust the odds ratio between the evaluators for each item. S-CVI/Ave was determined by taking the sum of the I-CVIs/the total number of items. Total SCVI/Ave of the initial questionnaire was 0.901 (acceptable value is 0.9). The minimum accepted value of CVR based on Lawshe’s CVR table was calculated as 0.80 to 0.78 for the remaining items. ^14^

### The Sixth Stage: Exploratory Factor Analysis (EFA) Used to Measure Construct Validity

In order to determine the validity of tool, we performed item analysis with 30 participants prior to construct validity. Based on the calculated alpha coefficient, 11 items were omitted because of item-total correlation coefficient<0.3. There was no overlap in this section between the two items (correlation coefficient>0.7) (the item was deleted if the inter-item correlation was more than 0.7). 

The sample size should be at least three times more than the number of items.12 The initial questionnaire contained 85 items (before item analysis). So, it was given to 255 postnatal women (85×3=255), who were selected by convenience sampling. On the other hand, 15 women who participated in the first two months after delivery had no sexual function, so the initial questionnaire was completed by 270 subjects. Kaiser-Meyer-Olkin (KMO) test and Bartlett’s Sphericity test were used. KMO values between 0.8 and 1 indicate the adequacy of sampling and Bartlett’s test shows the suitability of data for factor analysis (P=0.000). If the P value is <0.05, factor analysis is a good technique. ^16^ The inclusion criteria for participants in this phase consisted of being Iranian and being in the time period between ten days to first year postpartum with perineal injuries. Women with a history of medical or mental illnesses or those with a disabled child were excluded from the study. Eligible participants had referred to two urban health centers No. 1 and 3 in Mashhad. After completing the questionnaires, the data were analyzed by SPSS software, version 25.

**Figure 2:**
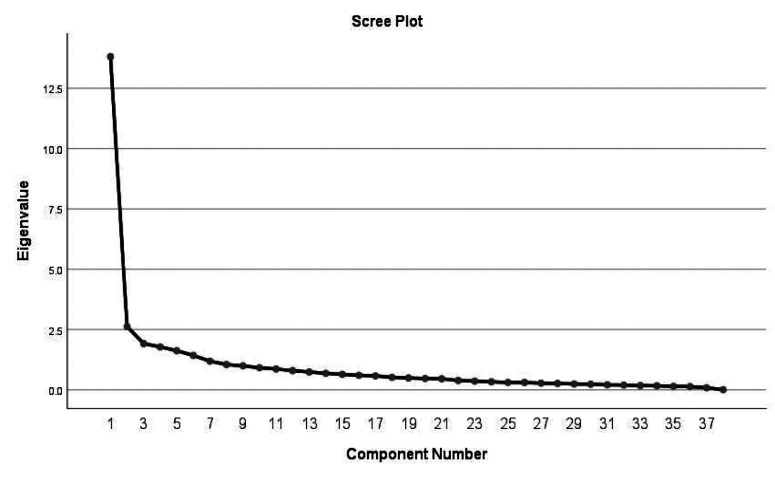
Determination of the Number of Factors of women’s recovery of postnatal perineal injuries questionnaire.

In this study, principal component analysis (PCA) was used to reduce a large dimension of data to a relatively smaller number of factors. The methods of scree plot, total variance explained, and Kaiser’s Criterion were simultaneously used to determine the dimensions of the recovery of postnatal perineal injuries. ^17^ In this analysis, the factor loadings >0.3 served as the minimum factorial load needed to maintain each item in the factor extracted from factor analysis. The final factors were extracted by Varimax rotation. Eight factors with an eigenvalue>1 and three with an eigenvalue >2 remained in this study (Figure 2). 

### The Seventh Stage: Evaluating the Items by Cronbach’s Alpha, Theta Coefficient and Intraclass Correlation Coefficient (ICC) 

*Reliability*

Internal consistency is always equated with Cronbach alpha. However, alpha coefficient is influenced by the sample size ^18^ , so Theta coefficient was calculated through the following formula: 

$\theta =\frac{N}{\mathrm{N-1}}[1-\frac{1}{{ƛ}_{1}}]$

In this formula, N is the number of questions and ƛ1 is the highest largest eigenvalue. ^19^ The calculated internal consistency for the items was high having Cronbach alpha and Theta Coefficient of >0.70. To determine the stability, we performed test-retest and 30 eligible postnatal women completed the final questionnaire in two stages with an interval of two weeks and the ICC of questionnaire was calculated. The range of ICC was between zero and 1. ^20^ Cronbach alpha, theta and ICC coefficient were calculated for each domain because the women’s recovery of postnatal perineal injuries questionnaire (WRPPIQ) is a profile and the scores of each domain is calculated separately. ^14^

### The Eighth Stage: Optimizing the Scale Length 

The length of the questionnaire was determined based on the Cronbach’s alpha coefficient. ^12^ Therefore, the research team decided to exclude the items that decreased the alpha coefficient or had an item-scale correlation <0.3. In this stage, no item was deleted.

### Ethical Considerations

The ethics committee of Mashhad University of Medical Sciences approved the study protocol (ethical code: IR.MUMS.REC.1395.568). In order to consider ethical issues, the first researcher explained the purpose of this study to the participants and after obtaining written consent, asked them to participate in the study. Also, information about confidentiality, anonymity and the right to withdraw from the study was given to the participants.

## RESULTS

Out of the 270 (100%) Iranian patients studied, 157 (58.1%) women were primiparous and 113 women (41.9%) were multiparous. 240 (88.9%) participants were housewives. Their mean age was 27.5±5 years. Some basic characteristics of the participants are presented in Table 1.

**Table 1:** Basic characteristics of the participants (n=270)

Variables	N (%)
Education	
≤Diploma	206 (76.3)
>Diploma	64 (23.7)
Parity	
First	157 (58.1)
Second	75 (27.8)
Third	30 (11.1)
Fourth	8 (3)
Mode of birth	
Spontaneous	238 (88.1)
Vacuum extraction	32 (11.9)
Degree of laceration	
First and second degree lacerations (+Episiotomy)	230(85.2)
3+4rd degree (sever tears)	40(14.8)
Days since birth	
10-90	128 (47.4)
91-180	52 (19.3)
270-181	53 (19.6)
271-365	37 (13.7)

According to the results of the qualitative phase, the initial questionnaire included 144 items (item pool included 202 and after revision by the research team, it was reduced to 144 items) in four categories of: “normalization of body function”(67 items), “moving to subjective well-being”(37 items), “moving to interactive empowerment” (28 items), and “promotion of health in the shadow of spiritual resilience (12 items). 

Normalization of the body function was conceptualized by objective and subjective experiences of the participants, in which changes in their body function occurred over a range of irregularities to regularity. “Moving to subjective well-being” was defined as level of happiness and satisfaction of the participants in their lives. “Moving to interactive empowerment” was defined as experiences and understanding of the participating women about their ability to take part in the social activities and interacting with others after birth. “Promotion of health in the shadow of spiritual resilience” was defined as the context for previous three categories; the participants commented that their tolerance increased in the shadow of religious and spiritual beliefs in physical (normalization of the body function), emotional (moving to subjective well-being), and social (moving to interactive empowerment) aspect of their life.

**Figure 3:**
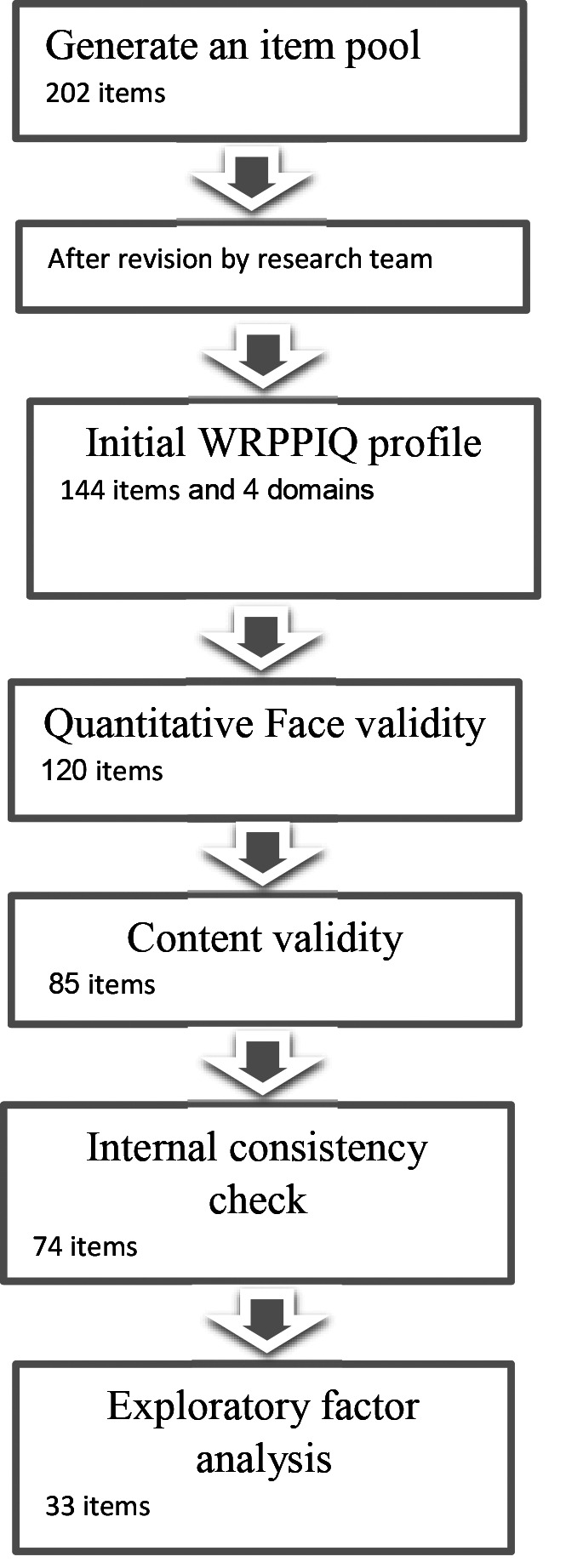
Item reduction in the process of women’s recovery of postnatal perineal injuries questionnaire psychometric analyses

In measuring quantitative face validity, 24 items were removed. Following calculation of CVI and CVR to evaluate the content validity, 35 items were removed. Therefore, the remained 85 items entered into item analysis. Eventually, 85 items were developed in four dimensions including normalization of the body function (30 items), moving to subjective well-being (29 items), shifting to interactional empowerment (18 items), and promoting health in the shadow of spiritual resilience (8 items). Figure 3 presents item reduction in the process of psychometric analyses.

In order to measure validity, we calculated the Cronbach’s alpha for each domain separately; 11 items were removed and finally 74 items entered into exploratory factor analysis. The initial Cronbach’s alpha measured for the domain was >0.83. After the questionnaires were completed by 270 women, factor analysis was performed. In the first stage of sampling with 255 samples, it was not possible to do Kayser-Meier-Alkin sampling adequacy test and Bartlett’s test; as 15 women were in the first month after delivery and had no sexual intercourse, they were unable to answer the items of 15-19, so it was added to 255 and the final sample reached 270 cases. Measurement adequacy was confirmed with KMO=0.835 and Bartlett’s spherical test significance was confirmed (X2=121155.516, P=0.001). Based on the correlation matrix and communalities statistics, gradual elimination of the items with correlation less than 0.4 was performed; 36 items were removed and then the factor analysis was continued with 38 items. Three factors were identified based on a rational and combination interpretation of the factors and the Scree plot (Figure 1).

These three factors revealed 47.420% of the total variance in the analysis. Next, factor analysis was performed for 38 items loading for derivation of three factors.

Table 2 shows the initial eigenvalues and the percentage of variance explained by the components extracted in the rotated matrix (PAC extraction method and orthogonal rotation with the Varimax)

**Table 2:** Factors with initial eigenvalues and percentage of total variance explained

Component	Eigenvalues (before rotation)			Rotation Sums of Squared Loadings		
	Total	Variance %	Cumulative %	Total	Variance %	Cumulative %
1	13.595	35.776	35.776	7.621	20.055	20.055
2	2.319	6.103	41.879	5.609	14.761	34.816
3	2.106	5.542	47.420	4.790	12.605	47.420

After the Varimax rotation, items with correlation less than 0.4 were removed. Therefore, three items were not loaded at all the factors, and the correlation of two items were less than 0.4, so all the five items were removed .When the factors were identified, they were named based on their items, especially the items with the highest factor loading and also knowledge gained from qualitative part of the study ^20^ (Table 3). 

**Table 3:** Exploratory factor analysis: Results for postnatal recovery in women following perineal Injuries (WRPPIQ)

Items	Domain	Factor 1	Factor 2	Factor 3
	Evidence of wellness (18 items)			
1. I need help for walking.		0.677		
2. I cannot sit or get up.		682.0		
3. I cannot stand up.		0.521		
4. I cannot walk.		0.677		
5. My perineal wound have an unpleasant smell.		0.665		
6. My stitches are red and swollen.		0.587		
7. I bleed while excreting.		0.489		
8. I cannot control the release of my urine.		469		
9. I feel pain during sex.		0.436		
10. I never reach orgasm.		0.730		
11. I am not sexually satisfied.		0.665		
12. Prescription of drugs reduced my lactation, so my baby is not gaining weight.		0.714		
13. Incision pain prevented me from breast feeding, so my baby is not gaining weight.		0.567		
14. My health is lower than before pregnancy.		0.603		
15. My physical problems take longer to heal (fatigue, low back pain, headache, constipation).		0.536		
16. My need for rest and care is more compared to healthy women.		0.424		
17. My medical visits are more than women with normal deliveries.		0.487		
18. The lack of libido has worried me.		0.426		
	Positive mood and emotional changes (9 items)			
19. I feel depressed.			0.524	
20. I get angry sooner than before.			0.563	
21. I am ashamed of inability to control gas passing.			480	
22. I feel like I have no love for my child.			0.750	
23. I cry for no reason.				
24. I often fight with my husband.			0.563	
25. I am not anxious.			0.789	
26. As always, I love my life.			0.750	
27. I have got a sense of renewed energy.			0.703	
	Independence and support (6 items)			
28. I do not intend to meet with family and friends.				488.
29. Due to the complications of wound healing, I can not attend ceremonies and parties.				0.786
30. I have regained the ability to manage my personal hygiene.				0.602
31. I regained my abilities through receiving family support.				0.807
32. The notification of health center and hospital helped more rapid healing of my perineal wound.				0.817
33. Access to medical services (health centers or hospitals) facilitated my postpartum recovery.				0.486

WRPPIQ: Women’s recovery of postnatal perineal injuries questionnaire

Cronbach’s alpha coefficients was calculated (n=270), indicating excellent internal consistency for WRPPIQ tool. Cronbach’s alpha coefficients for each evidence of wellness, positive mood and emotional changes, as well as independence and support were 0.92, 0.80 and 0.83, respectively. The results of the test-retest reliability showed that there was an excellent ICC (n=30) between baseline and 2 weeks for WRPPIQ tool (95% CI) (Table 4). No item was deleted due to favorable Cronbach’s alpha. 

**Table 4:** Cronbach’s alpha coefficients, Theta and ICC values for each domain of WRPPIQ

Scores obtained	α	Ɵ	ICC absolute agreement	CI (95%)	Mean±SD
Factor 1	0.92	0.91	0.93	0.728-976	66.77±10.85
Factor 2	0.80	0.92	0.95	0.895-0.977	30.51±7.72
Factor 3	0.83	0.94	0.64	0.664-0.824	23.26±3.55
Total	0.93	0.92	0.94	0.841-0.980	121.07±17.29

### Description of the Questionnaire

This is a self-report questionnaire scale developed in the Persian language which aimed to assess postnatal recovery in women following perineal trauma. The 33 items in three domains were scored using Likert scale responses from strongly agree=1 to strongly disagree=5. A set of items was scored reverse (include 25, 26, 27, 28, 31, 32, and 33) as strongly agre =5, Agree =4, Undecided=3, Disagree=4 and strongly disagree=1. This scale is a profile and the scoring is done for each domain. Higher scores in each domain mean better recovery in the same dimension. In this questionnaire, evidence of wellness, positive mood and emotional changes and independence and support have a minimum score of 18, 9, 6 and maximum score of 90, 45, 30, respectively.‎ 

### Feasibility 

The mean time to complete the questionnaire in the test was 5.6 minutes (ranged from 3 to 10 minutes) and the rate of non-response in all options ranged from zero to two percent.

### Scoring System of WRPPIQ

Standardization of the scores makes it possible to better understand and compare the scores obtained. Linear transformation formula was used to standardize the score ofthis questionnaire. ^15^ According to the following formula, the scores of this questionnaire were normalized. For example, in the 5-point Likert scale, each item had scores of 1 to 5, so to calculate the percentage score of subscale 3, the maximum score is 30 and the minimum score is 6 because it has 6 items. The scores obtained in each factor of questionnaire are presented as follows (without considering the weight of items):

**Evidence of wellness:** $\mathrm{Score Subscale1}=\frac{\mathrm{raw score obtained-18}}{\mathrm{90-18}}\times 100$

**Positive mood and emotional changes:** $\mathrm{Score Subscale2}=\frac{\mathrm{raw score obtained-9}}{\mathrm{45-9}}\times 100$

**Independence and support:** $\mathrm{Score Subscale3}=\frac{\mathrm{raw score obtained-6}}{\mathrm{30-6}}\times 100$

## DISCUSSION

WRPPTQ is a valid and reliable tool in postnatal recovery in women following perineal injuries. This questionnaire consists of 33 items distributed across three domains. It consists of closed-ended questions with options carrying physical, psychological, and social domains of recovery meanings. EFA revealed a 3-factor structure which indicates the multi-dimensionality of the constructs of WRPPIQ. This scale is an original and unique tool for assessment of postnatal recovery. 

In the present study, postpartum recovery constructs were extracted, and the items in the tools were developed based on the constructs of the concept of recovery structures. In the qualitative phase, the meaning of postpartum recovery was explained as evidence of wellness, positive mood and emotional changes as well as independence and support.

The fourth construct in qualitative analysis was “promoting health in the shadow of spiritual resilience”, which was not identified as a separate factor in EFA. In the qualitative analysis, of course, this construct was presented as the bedrock and background for the three other factors. Because the participants did not express this issue directly in their experiences, they acknowledged it in response to the probing questions as a basic component that facilitates their physical status and produces inner peace and helps them to be adapted with the problems in the recovery process. They also stated if there was no tolerance because of love for life and children or because of the reliance on the light of spirituality and Imams, they would never have been able to follow this path, so the spirituality and faith were the other dimensions of the analysis. In a qualitative study on 32 Canadian postpartum women who were in their first month after delivery, the physical issues was described as the most important domain in recovery, but the participants did not refer to sexual dysfunction and reduced breastfeeding and other dimensions of postnatal recovery. ^21^ The difference in the results of the Canadian study with the present research is related to the time of the study; the experience of the participants in the Canadian study was explored just in the first month postpartum in which women mainly suffer from physical pain, whereas in our study the experience of women was sought till one year after childbirth. Given that the sexual relationship is started in the second month postpartum, sexual well-being was also experienced as one dimension of postnatal recovery in our study. This issue has been argued as a dimension of health and wellbeing in pregnancy as well. ^22^ In another study conducted on 12 postpartum women with severe perineal trauma in Australia, ^23^ the theme of “they lived happily ever after” emerged, which refers to the emotional problems of these women. However, the focus of this study was not on postnatal recovery, so despite the similarity in the expression of emotional issues, no description has been presented regarding the return of happiness.

In a phenomenological study on 10 Australian women with perineal injuries, the theme of “resignation” was emerged. In this regard, the participants stated that they avoided group activities due to severe physical and urinary problems after birth. ^24^ This study also did not investigate postnatal recovery, so other dimensions of recovery including recovery of social status was not reported in this study. However, in the present study, in addition to physical and emotional recovery, social recovery, which refers to regaining the ability of interaction and communication and receiving support from family and community, as a facilitating factors for recovery, was emerged.

In a cross-sectional study on 183 Korean mothers, the participants reported that the support they received from the family and community was effective on postnatal recovery and it was very helpful in accepting the new responsibilities, the role of motherhood, and the changes which occurred in their body. ^25^ It is noteworthy that social communication is effective in providing emotional and cognitive needs in health. ^26^ In this study, the theme of “health promotion in the shadow of spiritual resilience”, which emerged from qualitative data analysis was not identified as a factor in EFA. Probably, the reason might be mathematical because the researcher noticed that the participants generally chose choices 2 (Agree) and 3 (Undecided) when they answered the questions related to this part, so no variance was found between the answers. A common limitation in factor analysis is related to the variance of the method; this limitation is identified in factor analysis when it becomes small due to homogeneous samples. ^27^ Therefore, it is necessary to replicate this research at different levels of religious and spiritual attitude.

In this study, like other validation studies, to develop clinical instruments in Iran, ^28^ we had no similar tools for comparison. Swedish Postoperative Recovery profile (PRP) had 19 items, and five domains including Physical Symptoms (Pain, Nausea, Appetite changes, Fatigue, Sleeping difficulties With 5 Items), Physical Function (Gastrointestinal function, Bladder function, Mobilization, Muscle Weakness And Sexual Activity With 5 items), mental status (Anxiety and worry, Feeling down, Feeling lonely and Difficulty in concentration with 4 items), social status (social activity - Dependence on others - Interest in surroundings with 3 items), and activity (Re-establishing everyday life and Personal hygiene with 2 items). ^29^ PRP has actually three social, physical, and psychological dimensions, whereas the signs and functions in WRPPIQ are integrated into one dimension of evidence of wellness. PRP is also a profile with the 4-point Likert scale, but it is not comparable with developed instrument in this study in nature and items. This study is the first attempt to evaluate the women’s recovery following perineal injuries after birth; therefore, it is recommended to be reassessed in terms of psychometrics in different cultures and different parts of Iran and using a random sampling method, if it is possible.

One of the limitations of the present study was convenient sampling and selection of a minimum sample size (three cases for each item) for EFA that was inevitably accepted due to the low number of patients with severe perineal injuries. However, indicators of sample size adequacy for factor analysis were found to be satisfactory. Also, the study was limited by the cross-sectional nature of the data, which did not capture the dynamics of recovery. The response bias due to asking the participants to self-report the answers to questions was another limitation of this study. However, all issues of minimum sample size, cross sectional nature of the data and also response bias have been reported in other validation studies to develop clinical instruments as well. ^30 - 32^

The strength of this study was designing a specific and multi-dimensional tool for postpartum recovery of women, especially those with severe perineal injuries. It can develop their care program and follow-up through facilitating the health assessment of these women. Development of specific tools helps to improve the quality of the midwifery care by the healthcare providers. ^33^

## CONCLUSION

The results of this study showed that the WRPPIQ has acceptable face validity, content validity, construct validity, internal consistency, and stability reliability in postnatal women with perineal injuries and can be used to evaluate the status of postnatal recovery. It is recommended that healthcare providers do not focus only on physical dimension of health in women with perineal trauma and pay attention to emotional and social dimensions of health as well. 
